# Development and Characterization of Dual-Platform Lyophilized Plasma-Based Quality Control Materials for Hepatitis C Virus Antibody Testing

**DOI:** 10.3390/diagnostics16121813

**Published:** 2026-06-12

**Authors:** Wipawee Thanyacharern, Wanvisa Treebuphachatsakul, Supaporn Suparak, Nam K. Tran, Napaporn Apiratmateekul

**Affiliations:** 1 Reference Materials and Innovation Research Unit for Medical Laboratory, Faculty of Allied Health Sciences, Naresuan University, Phitsanulok 65000, Thailand; wipaweeth65@nu.ac.th (W.T.); wanvisab@nu.ac.th (W.T.); 2 Department of Medical Sciences, Ministry of Public Health, Nonthaburi 11000, Thailand; supaporn.su@dmsc.mail.go.th; 3 Department of Medical Technology, Faculty of Allied Health Sciences, Naresuan University, Phitsanulok 65000, Thailand; 4 Computational Pathology and AI Center of Excellence (CPACE), School of Medicine, University of Pittsburgh, Pittsburgh, PA 15213, USA; trannk@upmc.edu

**Keywords:** hepatitis C virus, rapid diagnostic test, plasma-based material, quality control

## Abstract

**Background/Objectives:** Reliable quality control (QC) materials are essential for maintaining the analytical performance of hepatitis C virus (HCV) screening assays. Rapid diagnostic tests (RDTs) are widely used for point-of-care HCV screening; however, standardized plasma-based internal quality control (IQC) materials compatible with both rapid tests and automated immunoassays remain limited. This study aimed to develop and evaluate plasma-based QC materials applicable to multiple anti-HCV RDTs and automated immunoassays. **Methods:** QC materials were prepared from pooled HCV-positive plasma at strong-positive, weak-positive, and negative levels in liquid and lyophilized formats. Lyophilized preparations were produced with and without trehalose, while liquid samples were prepared with and without a stabilizer. Performance was evaluated using five anti-HCV RDT kits and the Elecsys Anti-HCV II automated immunoassay platform. Stability was assessed under accelerated temperature conditions (45 °C for 28 days) and long-term storage (2–8 °C and 20–30 °C for six months). Signal trends were analyzed using linear regression (*p* > 0.05), and homogeneity was evaluated using one-way analysis of variance and Cochran’s C test. **Results:** All QC formulations demonstrated consistent qualitative reactivity across the evaluated RDT kits and stable responses on the automated immunoassay platform. Lyophilized plasma containing trehalose maintained stable cut-off index (COI) values during accelerated and long-term storage, with no significant time-dependent trends (*p* > 0.05). **Conclusions:** Trehalose-stabilized lyophilized materials demonstrated enhanced stability and acceptable homogeneity, supporting practical applicability under the tested storage conditions across the evaluated rapid tests, and within the evaluated moderate-to-high positive analytical ranges on the automated anti-HCV immunoassay platform.

## 1. Introduction

Hepatitis C virus (HCV) poses a major global health challenge, causing acute and chronic infections that may lead to serious complications such as liver fibrosis, cirrhosis, and hepatocellular carcinoma [1]. Early detection through reliable serological screening is essential for timely treatment and prevention of disease progression [2]. Rapid diagnostic tests (RDTs) are widely used for first-line HCV antibody screening, particularly in decentralized and resource-limited settings due to their simplicity and rapid turnaround time [3,4]. The national testing guideline for HIV, syphilis, HBV, and HCV issued by the Department of Disease Control, Ministry of Public Health, Thailand, recommends the use of rapid antibody tests or laboratory-based immunoassays as an initial step, followed by confirmatory RNA testing for diagnosis and linkage to care [5].

In parallel, clinical laboratories increasingly rely on automated immunoassay platforms, such as chemiluminescent immunoassays (CLIA), electrochemiluminescence immunoassays (ECLIA), and enzyme immunoassays (EIA), to achieve high-throughput screening, enhanced analytical sensitivity, and quantitative signal measurement [5,6]. In addition to routine patient testing, automated systems also play a critical role in reagent lot-to-lot verification, calibration monitoring, and detection of analytical drift over time. To ensure longitudinal comparability of results, particularly in high-volume laboratories, maintaining consistency across reagent lots is essential. Therefore, QC materials for automated platforms must demonstrate adequate stability and matrix characteristics suitable for reliable lot-to-lot validation, inter-run assessments, and long-term performance monitoring in accordance with quality management systems [7,8].

In practice, current HCV diagnostic algorithms integrate two distinct approaches: qualitative rapid tests and quantitative automated immunoassays. Across these two diagnostic platforms, QC materials should demonstrate suitability for both testing modalities to support analytical consistency during routine quality monitoring [7,9]. However, standardized, plasma-based IQC materials validated across multiple RDT brands and automated systems remain limited. Commercial quality control materials are commonly designed for individual analytical systems and may not be suitable for harmonized quality assessment across multiple RDT brands and automated immunoassay platforms. In addition, access to stable commercial QC materials may be limited in decentralized or resource-limited settings, particularly under elevated environmental temperatures. Many laboratories rely on in-house prepared QC samples, which often lack systematic characterization of homogeneity, stability, and traceability [7,10,11]. This variability may compromise long-term analytical reliability, particularly in decentralized settings or environments with limited temperature control [12].

According to ISO 15189:2022 (Medical laboratories—Requirements for quality and competence), laboratories are required to implement IQC procedures to monitor the validity of examination results and participate in external quality assessment (EQA) or proficiency testing programs to verify performance [8]. An essential characteristic of reliable QC materials is a plasma matrix that behaves similarly to patient samples across different testing systems [10]. Materials that differ substantially from patient specimens may lead to misleading performance assessments and may not reflect actual analytical behavior in clinical testing. Therefore, plasma derived from clinical specimens is generally preferred when preparing QC materials intended for use across both rapid tests and automated immunoassay platforms. To address these requirements, ISO 33405:2024 provides guidance for evaluating the homogeneity and stability of reference and quality control materials [13]. An important characteristic of QC materials intended for use across multiple analytical systems is commutability, which refers to the ability of processed materials to behave similarly to patient specimens across different assays [10]. Formal commutability assessment according to CLSI EP14 guidelines was beyond the scope of the present study. Therefore, the developed materials were evaluated primarily for practical applicability across selected RDTs and an automated immunoassay platform rather than formal inter-assay equivalence. In addition, many commercial anti-HCV quality control materials are designed for platform-specific applications and may not support harmonized quality assessment across multiple RDT brands and automated systems under shared storage conditions. Aligning with ISO 15189:2022 quality management requirements and ISO 33405:2024 analytical evaluation principles, this study aimed to develop and characterize dual-platform, plasma-based QC materials for anti-HCV antibody testing. Through homogeneity testing, regression-based stability modeling, and dilution–response characterization, we sought to establish analytically evaluated QC materials suitable for both RDTs and an automated immunoassay platform under the evaluated storage conditions.

## 2. Materials and Methods

### 2.1. Sample and HCV Rapid Test Kits

Human plasma samples used in this study were derived from archived fresh frozen plasma (FFP) units obtained from the Thai Red Cross Society. All units were collected through routine voluntary blood donation procedures using citrate–phosphate–dextrose–adenine (CPDA-1) as the anticoagulant-preservative and processed according to standard blood bank procedures. HCV-positive samples were reactive for anti-HCV and nonreactive for human immunodeficiency virus (HIV), hepatitis B virus (HBV), and syphilis according to routine blood donor screening procedures. No additional blood collection from donors was performed. The study was approved by the Naresuan University Institutional Review Board (COA number: 118/2023; IRB number: P1-0024/2566) and the Research Ethics Committee of the National Blood Centre, Thai Red Cross Society (COA number: NBC 4/2024). Prior to subsequent analytical procedures, samples underwent heat inactivation according to routine laboratory biosafety procedures to reduce potential infectious risk during specimen handling and processing [14].

HCV-positive QC material was prepared by pooling plasma from five certified FFP units classified as anti-HCV reactive and/or HCV RNA positive based on routine screening results. HCV-negative plasma units confirmed as non-reactive for anti-HCV and negative for HCV RNA were used for dilution and preparation of negative control material.

Five commercial HCV RDT kits were evaluated: STANDARD Q HCV Ab (SD Biosensor, Inc., Suwon, Republic of Korea), Bioline™ HCV Hepatitis C Virus Test Kit (Abbott, Wiesbaden, Germany), One Step Hepatitis C Virus Serum/Plasma Test (Wondfo, Guangzhou, China), OnSite HCV Ab Plus Rapid Test (Strip) (CTK Biotech, Poway, CA, USA), and HCV Rapid Test Cassette (Acro Biotech, Inc., Rancho Cucamonga, CA, USA). These kits are hereafter referred to as kits 1–5 throughout the study.

### 2.2. Preparation of QC Materials

Plasma QC materials were prepared in two formats—liquid and lyophilized—to evaluate stabilization strategies under different storage conditions. For the liquid format, three formulations were prepared: HCV-positive plasma diluted with HCV-negative FFP, HCV-positive plasma diluted with StabilZyme™ SELECT Stabilizer (Surmodics, Inc., Eden Prairie, MN, USA) in HCV-negative plasma, and HCV-negative plasma used as the negative control.

For the lyophilized format, pooled HCV-positive FFP was supplemented with trehalose to a final concentration of 250 mM (approximately 8.6% *w*/*v*), adapted from previously reported dried biological quality control material stabilization approaches utilizing trehalose as a stabilizing excipient [15], prior to dilution with HCV-negative FFP. HCV-negative FFP was also used to prepare matched negative control materials. Plasma aliquots were frozen at −70 ± 5 °C for 24 h prior to lyophilization. The dried materials were reconstituted with 0.75 mL phosphate-buffered saline (PBS) containing 0.1% Tween 20 before analysis.

### 2.3. Dilution Response Characterization for QC Level Selection

Serial two-fold dilutions of pooled HCV-positive plasma were prepared and analyzed using the Elecsys Anti-HCV II assay (Roche Diagnostics, Rotkreuz, Switzerland) to characterize the dilution–response profile. Dilutions were prepared using HCV-negative plasma as the primary matrix, with assay diluent added only when required to maintain analytical compatibility. Cut-off index (COI) values obtained across the dilution series were examined to identify stable signal regions above the assay cutoff. Dilution points showing non-linear signal response or potential high-concentration effects were excluded from QC level selection. Platform-specific QC levels were subsequently defined based on stable COI regions while maintaining consistent qualitative detectability across the evaluated RDTs.

### 2.4. Evaluation of Rapid Test Procedures

**Figure 1:**
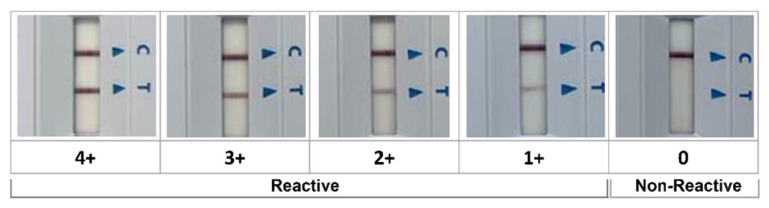
Visual grading scale for rapid anti-HCV test line interpretation. Representative examples of visual grading levels (0–4+) obtained from one commercial anti-HCV rapid diagnostic test are shown. Grades 1+ to 4+ were considered reactive, and grade 0 was considered non-reactive. C, control line; T, test line.

Five HCV RDT kits were evaluated according to the manufacturers’ instructions. All operators were trained to ensure consistent test performance and interpretation. Two independent readers visually graded the test line intensity using a standardized five-level scale (Figure 1): 0 (no visible line), 1+ (faint line, less than half the control line intensity), 2+ (approximately half the control line intensity), 3+ (greater than half the control line intensity), and 4+ (equal to the control line intensity). Qualitative concordance between observed results and predefined QC classifications was assessed. Homogeneity and stability were supported when complete concordance with the intended positive and negative classifications was observed.

### 2.5. Homogeneity Test

Homogeneity of the prepared QC materials was evaluated using a structured analytical approach. Ten tubes were randomly selected from each IQC batch and tested in duplicate within a single analytical run using the Elecsys Anti-HCV II system. Quantitative between-unit homogeneity was assessed using one-way analysis of variance (ANOVA) based on COI values, while within-unit variance consistency was evaluated using Cochran’s C test. In addition, qualitative homogeneity was evaluated using 10 randomly selected aliquots tested under identical conditions with the One Step Hepatitis C Virus Serum/Plasma Test. Qualitative assessment criteria included concordance of positive and negative results and visual comparison of test line intensity. Homogeneity was considered acceptable when no statistically significant between-unit differences were observed, and all qualitative results were concordant without visible variation in test line intensity, following ISO 33405:2024 [13].

### 2.6. Stability Testing

#### 2.6.1. Accelerated Stability Test

Accelerated stability was assessed by incubating plasma QC materials at 45 °C for 28 days. Baseline measurements were established on Day 0 prior to incubation. At predefined intervals (Days 7, 14, 21, and 28), aliquots were collected and stored at −20 °C until analysis. All samples were subsequently tested using the five anti-HCV RDT kits and the Elecsys Anti-HCV II automated immunoassay. Qualitative stability was evaluated based on the concordance of RDT classifications across all time points. For the automated assay, COI values obtained at each time point were analyzed using linear regression to assess time-dependent trends. The regression slope and associated *p*-value were used to evaluate potential degradation of the QC materials during the accelerated storage period.

#### 2.6.2. Long-Term Stability Test

Long-term stability was evaluated by storing plasma QC materials containing trehalose at 2–8 °C and 20–30 °C for up to 6 months, while reference control samples were maintained at −20 °C. Baseline measurements were obtained at Day 0 prior to storage. At monthly intervals, aliquots were retrieved and tested using the five anti-HCV RDT kits and the Elecsys Anti-HCV II automated assay. For the automated assay, each sample was analyzed in triplicate, and the mean COI value was used to represent the measurement at each time point to minimize the influence of potential analytical variation. Quantitative stability was evaluated using linear regression analysis of COI values as a function of storage time, where a non-significant regression slope (*p* > 0.05) was interpreted as indicating the absence of time-dependent degradation.

To further evaluate analytical performance stability on the automated platform, Levey–Jennings monitoring was performed using the 1:25 dilution QC material stored at 4 °C and 25 °C. The mean monthly COI values obtained from triplicate measurements were plotted against time. The mean and standard deviation (SD) used for the control chart were established from 20 baseline analytical runs.

### 2.7. Statistical Analysis

QC materials were analyzed using the Elecsys Anti-HCV II electrochemiluminescence immunoassay (ECLIA) on the cobas e411 analyzer (Roche Diagnostics, Rotkreuz, Switzerland). Reagents, calibrators, and controls were prepared according to the manufacturer’s instructions. Sample homogeneity was evaluated using one-way ANOVA, with calculated F-values compared against corresponding critical values. Homogeneity was considered acceptable when no statistically significant between-unit differences were observed. Temporal stability was assessed using linear regression analysis according to the model y = β_0_ + β_1_x, where y represents the COI value and x represents storage time. The regression slope (β_1_) and its associated *p*-value were used to evaluate time-related trends in COI values. A non-significant slope (*p* > 0.05) was interpreted as indicating the absence of detectable time-dependent trends under the evaluated conditions. The optimal QC formulation was selected based on regression-based stability results, qualitative concordance across the evaluated RDTs, and suitability under the evaluated storage conditions.

## 3. Results

### 3.1. Optimization of QC Level Selection for Rapid and Automated Platforms

QC materials were prepared at three concentration levels (strong-positive, weak-positive, and negative) and evaluated using five commercial HCV RDT kits. For rapid testing, the strong-positive level was defined at a 1:4 dilution, selected to consistently produce clearly visible reactive test lines across the evaluated kits while maintaining practical use of source plasma material. The weak-positive level was defined at a 1:25 dilution, selected to generate lower yet consistently detectable reactivity, enabling assessment of QC performance near the visual detection threshold. Negative control was prepared using HCV-negative FFP. Visual grading of rapid anti-HCV test line intensity was performed using a standardized five-level interpretation scale, as shown in Figure 1.

All three QC levels were tested across the five RDT brands. Qualitative reactivity patterns were concordant with the intended classification in all formats, although test line intensity varied among kits due to differences in antigen composition and signal chemistry. Weak-positive samples occasionally produced moderate intensity bands in certain kits, and strong-positive samples demonstrated kit-dependent variation in apparent intensity; however, qualitative classification remained consistent (Table 1).

**Figure 2:**
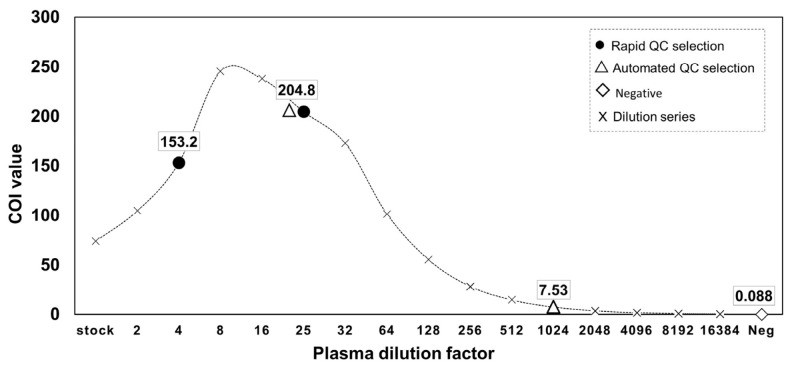
Dilution-dependent COI profile and automated QC level designation. Serial two-fold dilutions of pooled HCV-positive plasma were analyzed using the Elecsys Anti-HCV II assay to examine the COI response across the dilution series. Automated QC levels were selected from stable COI regions above the assay cutoff (COI = 1.0), while RDT QC levels were determined based on qualitative visual reactivity. COI, cut-off index; HCV, hepatitis C virus; QC, quality control; RDT, rapid diagnostic test.

Serial two-fold dilutions of pooled HCV-positive plasma were evaluated using the Elecsys Anti-HCV II automated chemiluminescent immunoassay to characterize the COI response across the dilution series (Figure 2). QC level designation was platform-specific because signal detection and interpretation differ between automated immunoassays and visually interpreted RDT kits. The dilution profile demonstrated non-linear signal behavior at higher antibody concentrations, followed by a progressive decline with increasing dilution. For the automated platform, QC levels were selected based on quantitative COI response rather than visual grading criteria. To avoid regions exhibiting non-linear high-concentration signal behavior, the 1:25 dilution (COI = 204.8) was selected as the high-positive automated QC level. Although the 1:25 dilution was categorized as weak-positive based on visual interpretation in RDTs, the corresponding COI values on the automated Elecsys Anti-HCV II platform remained within a high-positive analytical range. Therefore, the weak-positive designation in this study primarily reflected visually graded RDT performance rather than low-positive automated immunoassay characteristics near the assay cutoff. The 1:1024 dilution (COI = 7.53), positioned clearly above the assay cutoff (COI = 1.0), was selected as the low-positive automated QC level. The negative control (COI = 0.088) remained well below the cut-off.

These selections illustrate platform-dependent signal interpretation (visual grading versus quantitative COI measurement) while preserving a harmonized QC material framework across testing systems.

### 3.2. Homogeneity of QC Samples

Ten plasma QC units were randomly selected from each batch and tested in duplicate to evaluate between-unit homogeneity. Quantitative homogeneity was assessed using one-way analysis of variance (ANOVA) based on COI values. For strong-positive samples, the calculated F_cal was 2.416, indicating no significant between-unit variability. Similarly, weak-positive samples showed a low F_cal (0.842), which was lower than the corresponding critical value (F_crit = 3.020), supporting acceptable uniformity across units. Homogeneity was considered acceptable when calculated F-values did not indicate statistically significant differences between units (*p* > 0.05).

**Figure 3:**
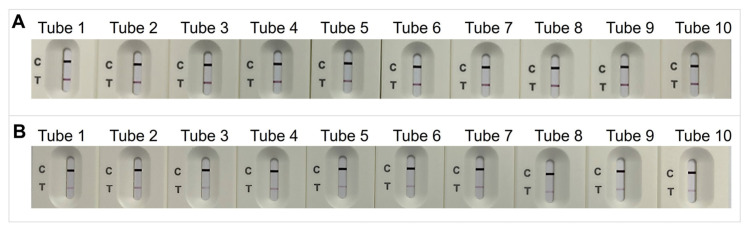
Representative homogeneity evaluation of lyophilized HCV QC materials using a commercial HCV RDT kit. (**A**) Strong-positive QC aliquots. (**B**) Weak-positive QC aliquots. All tested aliquots demonstrated concordant qualitative reactivity with comparable visual test line intensity.

Variance consistency was evaluated using Cochran’s C test. For strong-positive samples, the calculated C-value (C_exp = 0.430) was below the corresponding critical value (C_crit = 0.602), indicating acceptable variance consistency. For weak-positive samples, the C-value (C_exp = 0.478) was also below the critical limit (C_crit = 0.602), confirming that no individual unit contributed disproportionately to the overall variance. Representative homogeneity results obtained using one commercial HCV rapid diagnostic test (RDT) kit are shown in Figure 3, demonstrating concordant qualitative reactivity and comparable visual test line intensity among randomly selected QC units.

### 3.3. Accelerated Stability of the QC Samples

An accelerated stability study was conducted to evaluate the short-term thermal stability of the QC materials stored at 45 °C for 28 days. Changes in COI values over time were analyzed using linear regression, and the regression slope together with the corresponding *p*-value was used to assess time-related trends. Intermediate time points (Days 7, 14, and 21) were included in the regression analysis to evaluate temporal trends across the incubation period.

The regression analysis of different QC formulations under accelerated storage at 45 °C is summarized in Table 2. For liquid samples, weak-positive materials without stabilizer showed a significant negative slope (−1.864 COI/day, *p* = 0.02), indicating a decline in COI values during the incubation period. Liquid weak-positive samples containing stabilizers also demonstrated a significant decrease (−2.508 COI/day, *p* = 0.006), although the magnitude of decline differed between formulations. In contrast, strong-positive liquid samples showed small and non-significant slopes regardless of stabilizer addition.

Lyophilized samples generally exhibited smaller slopes than liquid formulations. However, lyophilized strong-positive samples without trehalose showed a statistically significant slope (−0.781 COI/day, *p* = 0.012), indicating measurable COI reduction under accelerated conditions. Lyophilized samples containing trehalose showed minimal changes in COI values throughout the 28-day period, with no statistically significant time-dependent trends observed. Lyophilized samples without trehalose also showed limited COI changes at the weak-positive level with non-significant slopes.

Based on these findings, the trehalose-containing lyophilized formulation was considered the most stable among the evaluated formulations and was therefore selected for subsequent long-term stability evaluation. The overall stability results obtained from accelerated testing at 45 °C are summarized in Table 3. Trehalose-containing lyophilized formulations demonstrated non-significant regression slopes (*p* > 0.05), indicating relatively stable COI responses during the 28-day incubation period. In contrast, statistically significant declines were observed in liquid weak-positive formulations and in lyophilized strong-positive samples without trehalose.

The QC materials used for stability evaluation were prepared in a separate production batch from the initial titration experiment. Consequently, the baseline COI values observed in the stability studies differed slightly from those obtained during titration (COI = 204.8), which are likely to reflect differences between independently prepared QC batches. Differences in regression trends and statistical significance observed between Table 2 and Table 3 reflect differences in analytical interpretation and summarized stability presentation rather than contradictory experimental outcomes. Minor variation in baseline COI values between independently prepared QC batches may also have contributed to differences in regression behavior during stability analysis.

**Figure 4:**
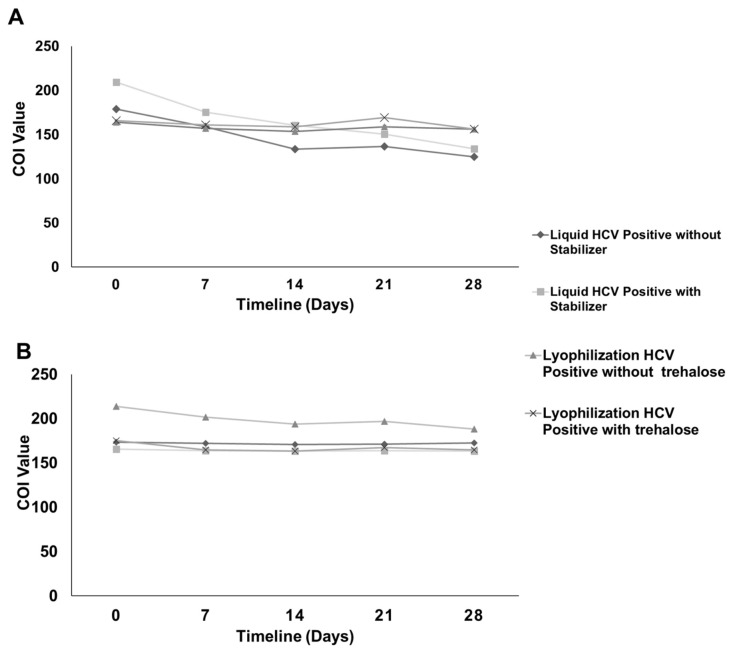
Temporal COI profiles of weak-positive and strong-positive plasma QC materials under accelerated storage at 45 °C. (**A**) COI values of weak-positive HCV plasma QC materials stored at 45 °C, comparing liquid and lyophilized formulations with and without stabilizers. (**B**) COI values of strong-positive HCV plasma QC materials under the same conditions. Trehalose-containing lyophilized formulations demonstrated relatively stable COI responses throughout the 28-day evaluation period. COI, cut-off index; HCV, hepatitis C virus; QC, quality control.

Across the evaluated conditions, all samples remained qualitatively reactive across the five anti-HCV RDT kits throughout the study period. Overall, these results indicate that lyophilized plasma QC materials, particularly those containing trehalose, maintained more stable COI responses under accelerated thermal stress, supporting acceptable analytical performance under the evaluated storage conditions. Graphical visualization of temporal COI trends across different formulations is presented in Figure 4.

### 3.4. Long-Term Stability of the QC Samples

Long-term stability of the plasma QC materials containing trehalose, at both strong-positive and weak-positive concentrations, was evaluated using five rapid anti-HCV test kits and the Elecsys Anti-HCV II system. Samples were stored at 2–8 °C and 20–30 °C for up to 6 months. Changes in COI values over time were assessed using linear regression analysis, with the regression slope and associated *p*-value used to evaluate time-related trends (Table 4).

Across both storage conditions, the regression slopes were small and not significantly different from zero (*p* > 0.05), indicating no significant time-dependent changes in COI values over the six-month period. Minor fluctuations in COI values were observed between Day 0 and Month 6; however, these variations were not statistically significant. Qualitative results obtained from all five rapid test kits remained reactive at all evaluated time points, supporting the observed stability of the plasma QC materials.

**Figure 5:**
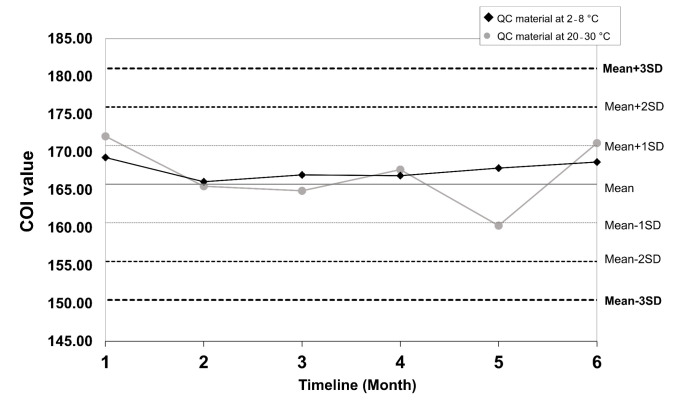
Levey–Jennings evaluation of long-term analytical performance of the 1:25 QC material stored at 4 °C and 25 °C. Means and SDs were established from 20 baseline measurements. Mean monthly COI values derived from triplicate measurements were plotted over the 6-month monitoring period. All results remained within ±3 SD limits, with no Westgard rule violations observed, supporting the analytical suitability of the QC material. COI, cut-off index; QC, quality control; SD, standard deviation.

To further confirm analytical performance stability on the automated platform, monthly monitoring was performed using Levey–Jennings analysis for the 1:25 dilution QC material stored at 4 °C and 25 °C (Figure 5). Means and SDs were established from 20 baseline analytical runs and mean monthly COI values derived from triplicate measurements were plotted over the 6-month monitoring period. All results remained within ±3 SD limits, with no Westgard 1–3 s rule violations observed, supporting the analytical suitability of the QC material.

## 4. Discussion

This study evaluated the optimal concentrations for QC samples across five HCV RDT kits based on test line color intensity. At the strong-positive level, Kit 1 exhibited the highest intensity (4+), followed by Kits 2, 4, and 5 (3+), while Kit 3 showed weaker intensity (2+). At the weak-positive level, Kit 1 again displayed the strongest intensity (3+), with Kit 5 following (2+), and Kits 2, 3, and 4 showing variable performance. A 1:4 dilution was determined to be more practical for QC preparation, as the 1:2 dilution required excessive stock resources, increasing costs and handling complexity. Differences in test line color intensity were attributed to variations in antigen composition among the kits. For example, Kit 3 included a broader range of antigens (Core, NS1, NS2, NS3, NS4, NS5), which could distribute antibody binding across multiple targets, potentially resulting in lower apparent signal intensity compared with kits containing fewer antigens. In addition to antigen composition, other factors such as antigen quantity, antigen type, and conjugate formulation could also influence test line intensity. Antigen design and calibration strategies are known to affect test sensitivity and specificity, particularly for samples containing low antibody concentrations [16]. The developed plasma-based QC materials were intended to complement rather than replace manufacturer-provided controls. Unlike many commercial controls that are optimized for individual analytical systems, the present materials were designed to support harmonized quality assessment across multiple RDT brands and an automated immunoassay platform under shared storage conditions.

For the automated Elecsys Anti-HCV II platform, dilution–response characterization enabled identification of stable COI regions above the assay cut-off for QC level designation. Notably, the 1:25 dilution—classified as weak-positive by visual grading in rapid tests—corresponded to a high COI region in the automated assay, emphasizing platform-dependent differences in signal interpretation. This discrepancy highlights the necessity of platform-specific QC level designation when harmonizing qualitative RDTs with quantitative automated immunoassay systems [17]. As shown in the dilution–response analysis, the automated immunoassay demonstrated nonlinear signal behavior at higher antibody concentrations. This characteristic may explain why the weak-positive QC level occasionally produced COI values comparable to or slightly higher than those of the strong-positive level. The lower COI values observed at dilutions below 1:16 may reflect nonlinear immunoassay behavior at very high antibody concentrations, potentially related to high-concentration effects, matrix interference, or partial assay saturation.

Following confirmation of acceptable homogeneity between QC units, the materials were subsequently subjected to accelerated and long-term stability evaluations to assess their analytical performance under different storage conditions. Long-term stability testing further confirmed the robustness of the trehalose-containing lyophilized format. Over 6 months at both refrigerated and room temperatures, regression slopes remained near zero with no statistically significant trends. Accelerated stability conditions at 45 °C preserved both quantitative COI stability and qualitative RDT reactivity. A temperature of 45 °C was selected to represent elevated environmental temperatures that may be encountered in tropical settings. In Thailand, the highest recorded ambient temperatures in 2024 ranged from approximately 43.0 to 44.5 °C during the summer season [18]. The dilution–response experiment also demonstrated that substantially higher dilutions produced COI values closer to the automated assay cutoff. However, these low-positive automated immunoassay levels were not included in subsequent stability studies. Therefore, stability findings on the automated platform should be interpreted within the evaluated moderate-to-high positive analytical range rather than cutoff-near low-positive conditions. Future studies should evaluate the stability performance of lyophilized QC materials at low COI levels near the assay threshold.

Furthermore, Levey–Jennings analysis demonstrated that the 1:25 QC material maintained analytical performance within ±3 SD limits throughout the monitoring period, with no Westgard 1–3 s rule violations observed [19]. Mean monthly COI values derived from triplicate measurements were plotted to monitor analytical performance over time. Notably, samples stored under refrigerated conditions (approximately 2–8 °C) demonstrated minimal variation throughout the 6-month monitoring period, supporting the suitability of refrigerated storage for maintaining long-term stability of the QC materials. These results indicate that lyophilization combined with trehalose improved QC stability under the evaluated accelerated and long-term storage conditions while maintaining acceptable analytical performance [15,20]. This characteristic may facilitate practical implementation of QC materials in routine clinical laboratories as well as decentralized or resource-limited settings, where stable and transportable quality control materials are essential for maintaining reliable diagnostic performance.

Previous studies have emphasized the importance of temperature control, lyophilization, and trehalose stabilization in preserving diagnostic reagent integrity [20,21]. However, prior investigations have primarily focused on short-term stability or single-platform applications and did not comprehensively evaluate long-term storage performance of plasma-based QC materials across multiple diagnostic systems. Compared with earlier reports, the present study extends existing knowledge by integrating long-term stability assessments with dual-platform evaluation. These findings provide a foundation for the analytical validation framework discussed below.

The evaluation framework was informed by ISO 33405:2024, which provides guidance for assessing homogeneity and stability of reference and QC materials [13]. Regression-based trend analysis, ANOVA-supported homogeneity testing, and longitudinal performance monitoring via Levey–Jennings charts collectively provide a structured analytical validation approach. This methodology also supports implementation of internal quality control requirements under ISO 15189:2022 by providing evidence-based validation of QC material suitability for routine laboratory monitoring. Although the developed QC materials demonstrated practical applicability across selected rapid diagnostic tests and the evaluated automated immunoassay platform, formal commutability assessment according to CLSI EP14 guidelines was not performed. Therefore, analytical equivalence between the processed QC materials and fresh patient specimens across different assay systems cannot be fully established. The present study should therefore be interpreted as a preliminary cross-platform feasibility evaluation rather than a formal commutability validation study. Further studies comparing the developed materials with native clinical samples across multiple analytical platforms would strengthen future commutability evaluation. Direct comparative evaluation against commercially available anti-HCV IQC materials was also not performed in the present study. In addition, homogeneity assessment for rapid diagnostic tests was based primarily on qualitative visual interpretation without quantitative image-based analysis or inter-rater agreement assessment. Although this approach reflected routine real-world RDT interpretation practices, future studies incorporating quantitative signal evaluation methods may further strengthen analytical characterization.

This study has limitations. Only five of the 81 Thai Food and Drug Administration (FDA)–approved HCV RDT kits were evaluated [22]. In addition, automated immunoassay performance was evaluated using a single analytical platform (Elecsys Anti-HCV II). The primary objective of this study was QC material development rather than comprehensive inter-brand performance comparison. Therefore, future studies should evaluate the applicability of the developed QC materials across additional automated platforms, RDT brands, kit lots, and real-world clinical workflows. Furthermore, extending this QC development framework to other infectious disease markers could enhance its scalability and clinical applicability. In addition, the QC materials were prepared from a single pooled plasma batch derived from five HCV-positive donors, which may not fully represent broader biological variability across clinical specimens.

Overall, trehalose-stabilized lyophilized plasma was successfully developed and demonstrated potential under evaluated conditions for anti-HCV antibody testing. Platform-specific QC level designation, combined with regression-based stability modeling and homogeneity assessment, establishes a structured and analytically sound framework for standardized plasma-based QC material development across the evaluated rapid diagnostic tests and automated immunoassay platform. Stability was demonstrated primarily for moderate-to-high positive analytical ranges on the automated platform, whereas low-positive automated immunoassay levels near the assay cutoff were not evaluated and require further investigation.

## Figures and Tables

**Table 1:** Quality control samples at different levels and in different formats tested using five HCV rapid test kits.

Kit	Strong Positive				Weak Positive				Negative	
	Liquid		Lyophilized		Liquid		Lyophilized		Liquid	Lyophilized
	− stab	+stab	−treh	+treh	−stab	+stab	−treh	+treh	Negative	Negative
1	4+	4+	4+	4+	3+	3+	3+	3+	0	0
2	3+	4+	3+	3+	1+	2+	1+	1+	0	0
3	2+	2+	2+	2+	1+	1+	1+	1+	0	0
4	3+	2+	3+	3+	2+	1+	2+	2+	0	0
5	3+	3+	3+	3+	2+	2+	2+	2+	0	0

Test line color intensity levels: 1+, 2+, 3+, and 4+ = reactive; 0 = non-reactive. Strong-positive and weak-positive classifications were defined based on visual band intensity for rapid diagnostic tests. −stab, without stabilizer; +stab, with stabilizer; −treh, without trehalose; +treh, with trehalose.

**Table 2:** Regression analysis of COI trends for different QC formulations under accelerated storage at 45 °C for 28 days.

Sample Type	QC Level	Regression Slope (COI/Day)	*p*-Value	HCV RDT Result (5 Kits)
Liquid without stabilizer	Strong positive	−0.039	0.390	Reactive
	Weak positive	−1.864	**0.020**	Reactive
Liquid with stabilizer	Strong positive	−0.066	0.100	Reactive
	Weak positive	−2.508	**0.006**	Reactive
Lyophilized without trehalose	Strong positive	−0.781	**0.012**	Reactive
	Weak positive	−0.208	0.314	Reactive
Lyophilized with trehalose	Strong positive	−0.271	0.262	Reactive
	Weak positive	−0.228	0.237	Reactive

Regression slopes represent the estimated daily change in COI values during accelerated incubation; *p*-values indicate the statistical significance of time-dependent trends based on linear regression. Regression slopes and *p*-values were derived from COI measurements obtained using the Elecsys Anti-HCV II automated immunoassay platform. Qualitative results from RDT remained reactive across all evaluated time points. Statistically significant *p*-values (*p* < 0.05) are shown in bold. COI, cut-off index; RDT, rapid diagnostic test; HCV, hepatitis C virus; QC, quality control.

**Table 3:** Regression-based stability assessment of trehalose-containing plasma QC materials selected based on favorable regression-based stability characteristics during accelerated stability screening, under storage at 45 °C for 28 days.

Temp (°C)	QC Level	COI (Mean ± SD)		Regression Slope (COI/Day)	*p*-Value	HCV RDT Result (5 Kits)
		Time Point (Day 0)	Time Point (28 Days)			
45	Strong positive	165.70 ± 1.41	161.30 ± 0.85	−0.089	0.478	Reactive
	Weak positive	166.55 ± 0.49	158.45 ± 0.07	−0.114	0.672	Reactive
	Negative	0.091 ± 0.004	0.091 ± 0.002	0.000	0.464	Non-Reactive

Regression slopes represent the estimated change in COI values per day during accelerated incubation; *p*-values indicate the statistical significance of time-dependent trends. COI values were obtained from QC materials prepared in a separate batch from the initial titration experiment. QC level classification was defined according to visual RDT reactivity, whereas COI values were measured using the Elecsys Anti-HCV II automated immunoassay platform. COI, cut-off index; RDT, rapid diagnostic test; HCV, hepatitis C virus; QC, quality control.

**Table 4:** Long-term regression-based stability assessment of trehalose-containing plasma QC materials stored at 2–8 °C and 20–30 °C for 6 months.

Temp (°C)	QC Level	COI (Mean ± SD)		Regression Slope (COI/day)	*p*-Value	HCV RDT Result (5 Kits)
		Time Point (Day 0)	Time Point (6 Months)			
2–8	Strong positive	158.40 ± 0.42	158.80 ± 0.14	0.017	0.368	Reactive
	Weak positive	169.65 ± 0.35	168.85 ± 0.21	−0.006	0.584	Reactive
	Negative	0.083 ± 0.001	0.084 ± 0.001	0.000	0.127	Non-Reactive
20–30	Strong positive	158.40 ± 0.42	161.55 ± 2.05	−0.018	0.764	Reactive
	Weak positive	169.65 ± 0.35	173.25 ± 2.90	−0.015	0.663	Reactive
	Negative	0.085 ± 0.001	0.083 ± 0.001	0.000	0.343	Non-Reactive

Regression slopes represent the estimated change in COI values over time; *p*-values indicate statistical significance of the time-dependent trend. Baseline COI values differ from the titration experiment because QC materials were prepared in an independent batch. QC level classification (strong-positive and weak-positive) was defined based on visual RDT reactivity, while COI values were measured using the automated immunoassay. COI, cut-off index; HCV, hepatitis C virus; RDT, rapid diagnostic test; QC, quality control.

## Data Availability

The original contributions presented in this study are included in the article. Further inquiries can be directed to the corresponding author.

## Abbreviations

The following abbreviations are used in this manuscript:

RDT	Rapid diagnostic test
QC	Quality control
HCV	Hepatitis C virus
IQC	Internal quality control
EQA	External quality assessment
Stabilizer	StabilZyme™ SELECT Stabilizer
ISO	International Organization for Standardization
DTS	Dried tube specimens
FFP	Fresh frozen plasma
SD	Standard deviation
